# Advancing *Eucalyptus* Genomics: Cytogenomics Reveals Conservation of *Eucalyptus* Genomes

**DOI:** 10.3389/fpls.2016.00510

**Published:** 2016-04-22

**Authors:** Teresa Ribeiro, Ricardo M. Barrela, Hélène Bergès, Cristina Marques, João Loureiro, Leonor Morais-Cecílio, Jorge A. P. Paiva

**Affiliations:** ^1^Linking Landscape, Environment, Agriculture and Food, Instituto Superior de Agronomia, University of LisbonLisboa, Portugal; ^2^Plant Cell Biotechnology Laboratory, Instituto de Biologia Experimental e TecnológicaOeiras, Portugal; ^3^Institut National de la Recherche Agronomique, Centre National de Ressources Génomiques VégétalesCastanet-Tolosan, France; ^4^RAIZ, Instituto de Investigação da Floresta e PapelAveiro, Portugal; ^5^Centre for Functional Ecology, Department of Life Sciences, University of CoimbraCoimbra, Portugal; ^6^Department of Integrative Plant Biology, Instytut Genetyki Roślin, Polskiej Akademii NaukPoznań, Poland

**Keywords:** BAC-landing, CCR1, ROP1, FISH, *Eucalyptus*, heterochromatin, transposable elements

## Abstract

The genus *Eucalyptus* encloses several species with high ecological and economic value, being the subgenus Symphyomyrtus one of the most important. Species such as *E. grandis* and *E. globulus* are well characterized at the molecular level but knowledge regarding genome and chromosome organization is very scarce. Here we characterized and compared the karyotypes of three economically important species, *E. grandis, E. globulus*, and *E. calmadulensis*, and three with ecological relevance, *E. pulverulenta, E. cornuta*, and *E. occidentalis*, through an integrative approach including genome size estimation, fluorochrome banding, rDNA FISH, and BAC landing comprising genes involved in lignin biosynthesis. All karyotypes show a high degree of conservation with pericentromeric 35S and 5S rDNA loci in the first and third pairs, respectively. GC-rich heterochromatin was restricted to the 35S rDNA locus while the AT-rich heterochromatin pattern was species-specific. The slight differences in karyotype formulas and distribution of AT-rich heterochromatin, along with genome sizes estimations, support the idea of *Eucalyptus* genome evolution by local expansions of heterochromatin clusters. The unusual co-localization of both rDNA with AT-rich heterochromatin was attributed mainly to the presence of silent transposable elements in those loci. The cinnamoyl CoA reductase gene (CCR1) previously assessed to linkage group 10 (LG10) was clearly localized distally at the long arm of chromosome 9 establishing an unexpected correlation between the cytogenetic chromosome 9 and the LG10. Our work is novel and contributes to the understanding of *Eucalyptus* genome organization which is essential to develop successful advanced breeding strategies for this genus.

## Introduction

Eucalypts (*Eucalyptus* L'Her) belong to Myrtaceae family and are predominantly native to Australia and to its North islands, where they occur in various habitats. Plants from this genus are highly diverse and display significant adaptability and phenotypic plasticity. They can be giant trees like *E. regnans* or shrubs and “mallees” like *E. pulverulenta* (Grattapaglia et al., 2012). The latest taxonomic revision (Brooker, 2000) of the eucalypts recognizes just over 700 species that belong to 13 subgenera including *Corymbia* K.D. Hill & L.A.S.Johnson (the bloodwood eucalypts) (Hill and Johnson, 1995), and *Angophora* Cav. (Ladiges, 1997). Most species belong to the subgenus Symphyomyrtus Schauer, which is divided in 14 sections (Brooker, 2000). From all of these sections only three are used in plantation forestry for industrial purposes: sect. Latoangulatae (*E. grandis* and *E. urophylla*), and sect. Exsertaria (*E. camaldulensis*) which grow in tropical and subtropical regions; and sect. Maidenaria (*E. globulus*) which grows in temperate regions (Grattapaglia et al., 2012). These species and their hybrids are among the world's leading sources of woody biomass and are the main hardwoods used for pulpwood and timber.

The main breeding programs in eucalypts worldwide are focused on improving profit from industrial pulp-wood plantations. Traditionally, key target traits are volume per hectare, wood density, and pulp yield. Secondary wood traits include the quantity or quality of extractives and lignin in the wood that affect the economic and/or environmental cost of pulping (Myburg et al., 2014). Wood properties and final uses are mainly determined by the relative abundance of the three major polymers deposited in the secondary cell walls of xylem cells, namely cellulose (40–50%), hemicellulose (25%), and lignin (25–35%) (Plomion et al., 2001). The identification of major genes involved in lignin biosynthesis provides the foundation for the development of biotechnology approaches to develop tree varieties with enhanced processing qualities (Carocha et al., 2015). The cinnamoyl CoA reductase (CCR) is a key enzyme that converts the hydroxycinnamoyl CoA esters into monolignols, the monomeric units that are incorporated into the lignin heteropolymer. For this reason the CCR gene has been considered a good candidate gene for molecular breeding for wood properties in *Eucalyptus* (Grattapaglia et al., 2012). The first cDNA encoding CCR was isolated from *E. gunnii* EguCCR (Lacombe et al., 1997) whereas in *E. grandis* were reported two genes, EgrCCR1 which has been allocated to the Linkage group (LG) 10 and EgrCCR2 allocated to LG 6 (Carocha et al., 2015). EgrCCR1 was found to be strongly and preferentially expressed in developing xylem, in agreement with its role in developmental lignin biosynthesis (Carocha et al., 2015). Another gene associated with cell growth, differentiation and developmental regulation, is the molecular switch Rho-related small GTP-binding protein EgROP1 or EguRAC1, wich is involved in cell differentiation during secondary xylem formation and might affect lignin composition and quality (Foucart et al., 2009).

The availability of *E. grandis* genome sequence (Myburg et al., 2014) together with increasingly powerful molecular technologies provide exceptional opportunities for whole-genome comparative and evolutionary studies across species of *Eucalyptus*. Although there is a huge amount of molecular resources for comparative *Eucalyptus* studies, including genetic linkage maps constructed with transferable microsatellites (Grattapaglia et al., 2015), QTL mapping (Gion et al., 2015), and microarray-based Diversity Array Technology (DArT) (Grattapaglia et al., 2011; Steane et al., 2011), the knowledge of these genomes at the cytogenomic level is very scarce. The correlation between linkage maps built with several molecular markers and physical chromosomes is not always direct, despite the continued addition of new markers and genes to previous maps. The assembled meta-chromosomes of the *E. grandis* reference genome sequence were numbered and oriented according to conventional linkage maps using a genome-wide framework of microsatellite and DArT markers (Bartholomé et al., 2015) even though the assigning to linkage maps was only inferred. Only few studies concerning chromosomal counting in several species (Matsumoto et al., 2000; Oudjehih and Abdellah, 2006) and genome size estimations of the economically most important species are available (Grattapaglia et al., 2012).

Comparative cytogenomic analysis is important to understand species evolution through the assessment of genetic divergence between them using genome organization knowledge (Lipman et al., 2013; Cai et al., 2014). Techniques such as fluorochrome banding (chromomycin, Hoechst, and DAPI) and fluorescence *in situ* hybridization (FISH) are exceptional molecular cytogenetic tools to reveal phylogenetic relationships among species in several crops (Maluszynska and Heslop-Harrison, 1993; Fuchs et al., 1998; Hajdera et al., 2003; Srisuwan et al., 2006), but also in trees (Ribeiro et al., 2008). The constitutive heterochromatin (AT-rich or GC-rich DNA) and rRNA genes are the most widely used FISH markers (Siljak-Yakovlev et al., 2014), however modern molecular cytogenetic studies benefit from BAC libraries. FISH using BACs probes has been used to enable successful genome assembly (Chamala et al., 2013; Myburg et al., 2014), to correct chromosome identity (Tuskan et al., 2006; Islam-Faridi et al., 2009), to map genes of interest (Mendes et al., 2011; Silva et al., 2015), and to serve as a supporting tool to select BAC clones for sequencing and tagging gene-rich regions in species with low sequence resources (Książkiewicz et al., 2013). BAC and fosmid clones have also been useful for the integration of genetic and physical maps (Mun et al., 2006) and in comparative genomics and assays of colinearity (Jenkins and Hasterok, 2007; Gu et al., 2009; Zhao et al., 2011; Yang et al., 2014).

In this study we characterized and compared the karyotypes of six eucalypt species belonging to the subgenus Symphyomyrtus: three with high economic importance, *E. grandis, E. globulus*, and *E. calmadulensis*, and three with ecological relevance, *E. pulverulenta, E. cornuta*, and *E. occidentalis*. To achieve this we used an integrative approach with estimation of genome sizes, fluorochrome banding, FISH mapping with rDNA markers, and BAC clones.

## Materials and methods

### Plant material

The plant material (Table 1) used in this study was obtained from the seeds collection of the herbarium of the Institute of Agronomy of the University of Lisbon, (Lisbon, Portugal) (LISI). Seedlings were maintained in a growth chamber (22 ± 2°C and photoperiod of 16/8 h of light and dark, respectively). Root tips for chromosome preparations and leaves for flow cytometric analyses were collected from these seedlings. Leaves of the internal reference standard for genome size estimations (*Solanum lycopersicum* “Stupické”) were obtained from seeds kindly provided by the Laboratory of Molecular Cytogenetics and Cytometry (Olomouc, Czech Republic), which were further germinated in pots in a growth chamber with the conditions described above.

**Table 1:** **Taxonomical classification of the plant material, native distribution, seeds provenance, and voucher numbers [LISI designation is in accordance to *Index Herbariorum* codes (Thiers, 2010)]**.

**Species**	**Subgenus**	**Section**	**Native**	**Source**	**Voucher**
*E. occidentalis* Endl.	Symphyomyrtus	Bisectae	Western Australia	ISA campus	LISI 364/2015
*E. cornuta* Labill.	Symphyomyrtus	Bisectae	Western Australia	ISA campus	LISI 369/2015
*E. grandis* W. Hill ex Maid.	Symphyomyrtus	Latoangulatae	Australia	ISA campus	LISI 365/2015
*E. globulus* Labill.	Symphyomyrtus	Maidenaria	Tasmania	ISA campus	LISI 366/2015
*E. pulverulenta* Sims	Symphyomyrtus	Maidenaria	New Sowth Wales	ISA campus	LISI 367/2015
*E. calmadulensis* Dehnh	Symphyomyrtus	Exsertaria	Australia	ISA campus	LISI 368/2015

### *Eucalyptus globulus* BAC libraries and selection of BAC clones for BAC landing

The *E. globulus* BAC libraries EGC-Ba and EGC-Bb were kindly provided by RAIZ Institute (Portugal). The BAC libraries were prepared by Clemson University Genomic Institute (CUGI), from young leaves collected on a elite *E. globulus* tree (coded TUGAL) grown at Espirra (Portugal), using the CUGI own protocol. Extracted high molecular weight gDNA was restricted with EcoRI (library EGC-Ba) and HindIII (library EGC- Bb) to obtain the appropriate partial digestion conditions (Paiva et al., 2011). For both libraries, pulsed-field gel electrophoresis (PFGE) size-selected restriction fragments were ligated to the EcoRI or HindIII digest pIndigoBAC536 vector. Ligation products were transformed into DH10B T1 phage resistant Escherichia coli cells (Invitrogen, Carlsbad, CA).

### Identification of clones harboring genes of interest

For each library the 36,864 BAC clones were reorganized into 3D-pools at the French Plant Genomic Center (CNRGV) following the procotol used by Paux et al. (2008). The genes of interest were identified by screening these *E. globulus* BAC 3D-pools by PCR, using specific primers for the *E. gunnii* Cinnamoyl CoA reductase gene (CCR) (EguCCR; X79566) (F:TGATGAGGTGAACCCAAGAGTA and R:TTTCT CCTGCAAGCTCTTGACA) (Rasmussen-Poblete et al., 2008) and for the *E. gunnii* Rho-related small GTPbinding protein (EguRAC1; DR410036) (F:AGCGCGTCG AGGTTCATCAA and R: TGGGGAAAGTGTTGCTGGTATA) (Rengel et al., 2009). Chloroplastic and mitochondrial sequences harboring BAC clones were identified as described in Paiva et al. (2011).

### BAC clones sequencing

Before sequencing, for each selected BAC clone the presence of the gene/loci of interest was rechecked by PCR on one isolated colony. BAC insert sizes of seleced clones were estimated on the electrophoretic profile of NotI enzyme digested BAC DNA fragments and resolved by PFGE (CHEF-DRIII system, Bio-Rad) in a 1% agarose gel in TBE buffer 0.5×, using the conditions described in Paiva et al. (2011). BAC DNA extractions, sequencing library generation, and sequencing were performed by Beckman Coulter Genomics SA. BAC inserts were sequenced by pyrosequencing using a Roche GS FLX Life Sciences instrument (Branford, CT, USA) (Margulies et al., 2005). The MIRA software (http://sourceforge.net/projects/mira-assembler/) was used to perform de novo assembly of the Roche GS FLX reads.

### Chromosome preparations

C-metaphases were induced by treating roots with a saturated solution of α-bromonaphthalene for 3 h at room temperature. Root tips were fixed in fresh ethanol to glacial acetic acid solution (3:1, v/v), and well-spread chromosomes were obtained using the squash technique according to Schwarzacher and Heslop-Harrison (2000) with the enzymatic mixture proper to woody species (Ribeiro et al., 2011).

### Fluorochrome banding

Slides were incubated in McIlvane buffer pH 7.0 + Mg^2+^ during 15 min followed by an incubation with Chromomycin A3-staining (CMA3) 0.5% during 90 min in the dark at room temperature. The slides were washed in McIlvane buffer pH 7.0 and mounted in 4′,6-diamidino-2-phenylindole (DAPI). DAPI banding patterns were fully revealed after FISH experiments.

### Fluorescence *in situ* hybridization (FISH)

The following probes were used for FISH: 35S rDNA unit of wheat (pTa71) (Gerlach and Bedbrook, 1979), 5S rDNA unit of *E. globulus*, and the *E. globulus* BAC clones EGC_Ba_P37H11 (BAC-CCR1), and EGC_Ba_P22O03 (BAC-RAC1). Clone pTa71 and BAC probes were isolated with NZYMiniprep (NZYtech) and labeled with digoxigenin or biotin using the Nick translation mix (Roche). The 5S ribosomal DNA probe was generated by PCR using forward and reverse primers (F:CCTGGGAAGTCCTCGTGTTG and R:CTTCGGAGTTCTGATGGGAT) in *E. globulus* DNA labeled with digoxigenin or biotin.

FISH with several probes was performed sequentially. First was performed the BAC “landing” according to Jenkins and Hasterok (2007) with minor modifications: 68% of stringency in all FISH experiments either in self-species or cross-species hybridization. After BAC-FISH metaphases were photographed and the slides were further washed with glacial acetic acid (3:1, v/v), 2xSSC (saline sodium citrate buffer), and increasing ethanol series before hybridization with the rDNA probes according to Alves et al. (2012).

Slides were analyzed with appropriate filters in the epifluorescence microscope Imager Z1 (Zeiss). Images were captured by the monochromatic camera AxioCam Hrm (Zeiss) through the AxioVision software (Zeiss) and assembled by Photoshop CC software.

### Karyotype measurements

Chromosomes were paired by FISH marks, AT-rich bands, and the relative position of the primary constriction, from the biggest to the smallest pair. Measurements of total length and arm ratio were made in chromosomes of five metaphases with the measurement module of the AxioVision software. The nomenclature of the chromosomes was according with Levan and colleagues recommendations (Levan et al., 1964): M (centromere at median point), m (centromere at median region), sm (centromere at submedian region), and st (centromere at subterminal region).

### Genome size estimation using flow cytometry

The genome size of 10–11 individuals of *E. pulverulenta, E. occidentalis*, and *E. cornuta* was estimated using flow cytometry following Galbraith et al. (1983). Briefly, 50 mg of leaves from the sample material and from the reference standard [*S. lycopersicum* “Stupické”; 2C = 1.96 pg; (Doležel et al., 1992)] were chopped with a razor blade in a Petri dish containing 1 ml of WPB (Loureiro et al., 2007). Due to the high levels of phenolic compounds present in *Eucalyptus* leaves, we significantly reduced the chopping intensity. The nuclear suspension was then filtered through a 30 μm nylon filter and nuclei were stained with 50 mg ml^−1^ propidium iodide (PI) (Fluka, Buchs, Switzerland), and 50 mg ml^−1^ RNase (Sigma, St Louis, MO, USA) was added to the nuclear suspension to prevent staining of double-stranded RNA. Samples were analyzed within a 5 min period in a Partec CyFlow Space flow cytometer (532 nm green solid-state laser, operating at 30 mW; Partec GmbH., Görlitz, Germany). At least 1300 particles per G1 peak were acquired per samples using the Partec FloMax software v2.4d (Partec GmbH, Münster, Germany) (Suda et al., 2007). Despite of the difficulty of analyzing *Eucalyptus* species using flow cytometry, the average CV value for the G1 peak was below 5% (mean CV value = 4.48% for *Eucalyptus* spp.; mean CV value for the standard = 3.12%).

The genome size in mass units (2C in pg; sensu Greilhuber et al., 2005) was obtained as follows: *Eucalyptus* sp. 2C nuclear DNA content (pg) = (*Eucalyptus* sp. G1 peak mean / reference standard G1 peak mean) ^*^ genome size of the reference standard. Differences in genome size among species were evaluated with a One-Way ANOVA (SigmaPlot).

## Results

### BAC identification and sequences analysis

The BAC library contaminations with chloroplastidial and mitocondrial sequences were respectively, 2.16% and 1.17% for the library EGC_Ba, and 0.20% and 0.77% for the EGC_Bb. The average size of the inserts for both libraries was estimated at 115 kb. Based on the number of clones of each library (36,864 clones for EGC_Ba and 36,864 clones for EGC_Bb) and the size of the *E. globulus* genome estimated at 1.09 pg/2C (530 Mb/1C) (Grattapaglia and Bradshaw, 1994), the genome coverage of both libraries is 11.2X, and the probability to find a BAC clone harboring a sequence of interest is 99.9% based on the algorithm of Clarke and Carbon (1976).

The 3D-pool approach developed at the French Plant Genomic Center (CNRGV) (Rasmussen-Poblete et al., 2008) revealed to be very efficient in the identification of BAC clones harboring the two genes of interest. Using the primers developed for *E. gunnii* Cinamoyl-CoA reductase (EguCCR, X79566) (Rengel et al., 2009) and *E. gunnii* GTPase RAC (EguRAC1, DR410036) (Rasmussen-Poblete et al., 2008) a total of eight and seven BAC clones were identified, respectively. Among these, the BAC clones EGC_Ba_P37H11 and EGC_Ba_P22O03, harboring respectively the EglCCR1 and EglRAC1 genes, were selected for sequencing (Table 2). For EGC_Ba_P37H11 93% of the 64,290 454-reads were assembled into 14 contigs (> 500 bp), with a final assembling size of 138.5 kb. For EGC_Ba_P22O03, also 93% of the 80,593 reads were assembled into 37 contigs (>500 bp), with an assembling size of 141.7 kb. The assembled size of EGC_Ba_P22O03 was similar to that obtained by enzymatic restriction. However, taking into account that the insert of EGC_Ba_P37H11, calculated by enzymatic restriction is about 100 kb, this means that some of the contigs (>500 bp) were overlapping each other.

**Table 2:** **Summary of the statistics of sequenced and assembled BAC clones**.

**BAC clone**	**Gene**	**Number of reads**	**% assembled reads**	**Contigs (>500 bp)**	**Assembled size (bp)**
EGC_Ba_P37H11	EglCCR1	64,290	93	14	138,584
EGC_Ba_P22O03	EglRAC1	80,593	93	37	141,706

The presence of both genes of interest was confirmed by Blastn against the non-redundant nucleotide database of NCBI. Besides, EGC_Ba_P37H11 and EGC_Ba_P22O03 sequences mapped as expected at the scaffold J of *E. grandis* genome (Myburg et al., 2014), in the region containing the *bona fide* EgrCCR1 (Egr.J03114) (Carocha et al., 2015) and the EgrGTPase-RAC (Eucgr.J02468). EGC_Ba_P22O03 and EGC_Ba_P37H11 sequences were deposited at GeneBank under the accession numbers KU727161 and KU727162, respectively.

RepeatMasker Web Server (http://www.repeatmasker.org/cgi-bin/WEBRepeatMasker) vs. open-4.0.5 was used to screen both BAC clone sequences against the DNA sequences in FASTA format against the Repbase-derived RepeatMasker library of repetitive elements (RMLib: 20140131) or against the Dfam database (Dfam: 1.3). The CG content was similar for both BAC clones, 39.20% and 40.92% for the EGC_Ba_P37H11 and EGC_Ba_P22O03, respectively. Contrary to that observed for clone EGC_Ba_P37H11 (only a 48 bp signal of a Ty1/Copia retroelement), the sequence of EGC_Ba_P22O03 revealed the presence of of two larger Ty1/Copia (total 2024 bp) and two Gypsy/DIRS1 (total 853 bp) retroelements and six DNA mutator transposon autonomous regulators, MuDR (357 bp). Moreover, EGC_Ba_P22O03 presented almost the double number of simple repeats (105, total of 3986 bp) of that of the EGC_Ba_P37H11 (55; total of 1823 bp) and also numerous (total 26) low complexity sequences not observed in EGC_Ba_P37H11. The presence of these repetitive elements identified by RepeatMasker could partially explain the high number of contigs (>500) observed for EGC_Ba_P22O03 BAC clone (37 contigs) when compared with that of obtained for EGC_Ba_P37H11 BAC clone (14 contigs).

### Karyotype analysis

**Figure 1:**
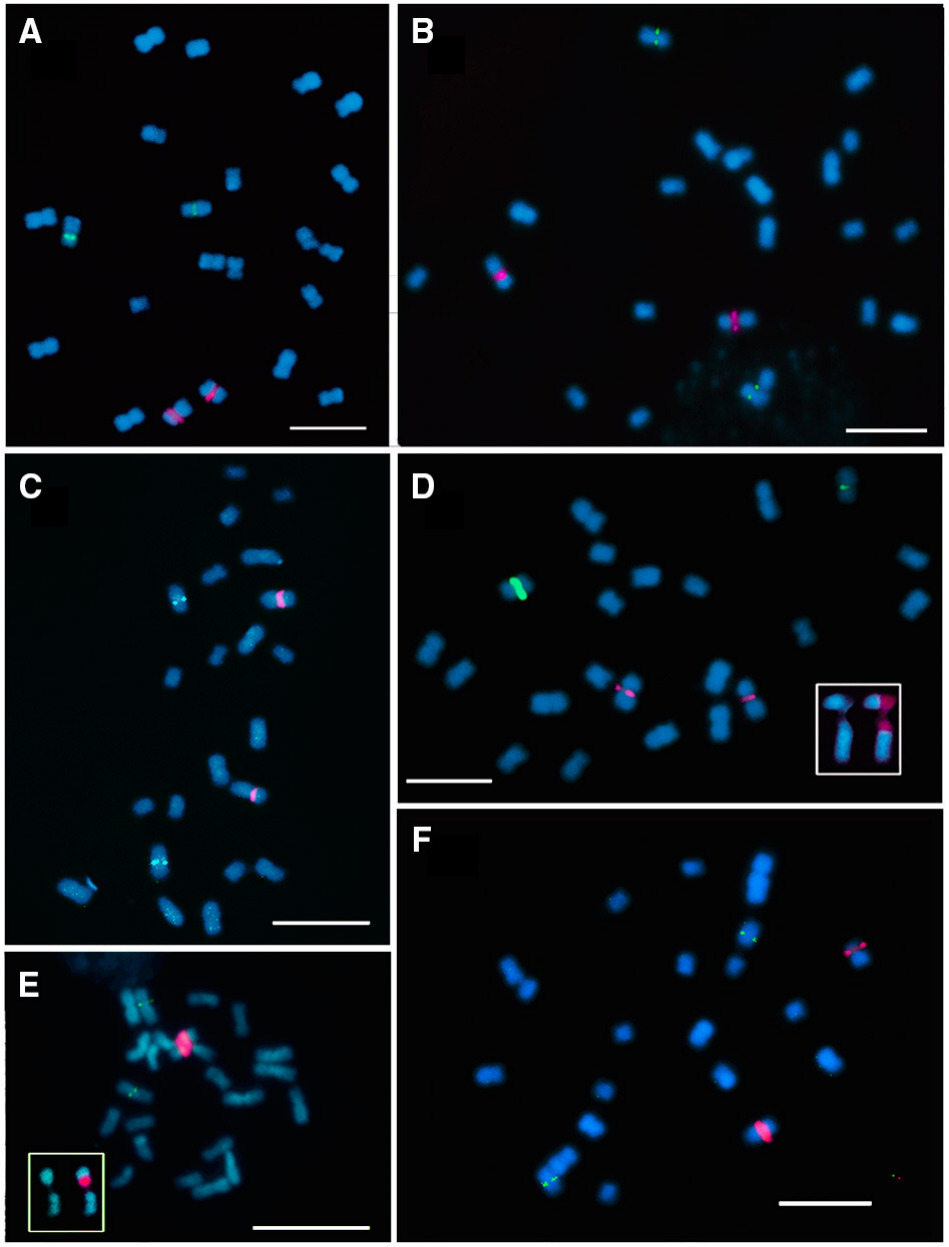
**Fluorescence *in situ* hibridization with 35S rDNA (red signal) and 5S rDNA (green signal) probes in metaphase chromosomes of *E. cornuta* (A); *E. occidentallis* (B); *E. camaldulensis* (C); *E. grandis* (D); *E. globulus* (E); *E. pulverulenta* (F)**. Insets showing the 35S rDNA locus (NOR) organization: **(D)** *E. grandis* pattern with decondensed domain in the secondary constriction and two condensed domains in both edges (red) of the NOR; Inset in **(E)** showing a decondensed domain in the secondary constriction and one condensed domain in the distal end (red). Bar = 5 μm.

All species studied presented 2*n* = 2*x* = 22 chromosomes (Figure 1). The mean length of the haploid set ranged from of the studied species is 23.24–20.04 μm in *E. grandis* and *E. pulverulenta*, respectively. All karyotypes were composed by small chromosomes with total lengths and arm ratios very similar among species, nevertheless, all chromosomes could be paired and identified and no overlapping was observed between contiguous chromosomes. The karyotypes were mainly composed by chromosomes with the centromere at median region (m) with few pairs with the centromere at median point (M). However, in the species of Bisectae section (*E. occidentalis* and *E. cornuta*) one or two pairs, respectively, presented the centromere at subterminal region (sm). In summary, the karyotype formula for *E. camaldulensis, E. grandis*, and *E. globulus* is 4M + 18m; for *E. pulverulenta* is 2M + 20m; and for *E. cornuta* and *E. occidentalis* is 2M + 16m + 4sm and 2M + 18m + 2sm, respectively (Table 3).

**Table 3:** **Karyotype measurements of *Eucalyptus* species**.

	**Section Bissectae**						**Section Exsertaria**			**Section Latoangulatae**			**Section Maidenaria**					
	***E. cornuta***			***E. occidentalis***			***E. camaldulensis***			***E. grandis***			***E. globulus***			***E. pulverulenta***		
**Chr**.	**Total length (μm) Mean ± SD**	**Arm ratio Mean ± SD**	**Type**	**Total length (μm) Mean ± SD**	**Arm ratio Mean ± SD**	**Type**	**Total length (μm) Mean ± SD**	**Arm ratio Mean ± SD**	**Type**	**Total length (μm) Mean ± SD**	**Arm ratio Mean ± SD**	**Type**	**Total length (μm) Mean ± SD**	**Arm ratio Mean ± SD**	**Type**	**Total length (μm) Mean ± SD**	**Arm ratio Mean ± SD**	**Type**
1	2.58 ± 0.17	1.13 ± 0.06	m	2.65 ± 0.20	1.20 ± 0.13	m	2.52 ± 0.12	1.06 ± 0.04	m	2.61 ± 0.13	1.19 ± 0.15	m	2.67 ± 0.18	1.05 ± 0.07	m	2.44 ± 0.15	1.16 ± 0.02	m
2	2.46 ± 0.08	1.04 ± 0.06	m	2.50 ± 0.10	1.13 ± 0.10	m	2.45 ± 0.04	1.08 ± 0.04	m	2.55 ± 0.09	1.04 ± 0.03	m	2.57 ± 0.09	1.04 ± 0.01	M	2.28 ± 0.07	1.01 ± 0.02	M
3	2.32 ± 0.05	1.18 ± 0.09	m	2.44 ± 0.08	1.17 ± 0.09	m	2.36 ± 0.05	1.26 ± 0.08	m	2.49 ± 0.08	1.26 ± 0.09	m	2.44 ± 0.08	1.25 ± 0.09	m	2.16 ± 0.06	1.06 ± 0.06	m
4	2.20 ± 0.07	1.13 ± 0.12	m	2.33 ± 0.07	1.45 ± 0.13	m	2.28 ± 0.06	1.40 ± 0.09	m	2.40 ± 0.08	1.03 ± 0.06	m	2.30 ± 0,07	1.02 ± 0.02	M	2.05 ± 0.05	1.09 ± 0.10	m
5	2.11 ± 0.06	1.00 ± 0.04	M	2.20 ± 0.07	1.11 ± 0.04	m	2.22 ± 0.07	1.03 ± 0.02	M	2.34 ± 0.08	1.19 ± 0.02	m	2.22 ± 0.07	1.04 ± 0.04	m	1.92 ± 0.07	1.19 ± 0.13	m
6	2.00 ± 0.05	1.15 ± 0.08	m	2.13 ± 0.09	1.73 ± 0.31	sm	2.15 ± 0.06	1.26 ± 0.02	m	2.22 ± 0.06	1.01 ± 0.02	M	2.15 ± 0.09	1.22 ± 0.05	m	1.81 ± 0.08	1.28 ± 0.09	m
7	1.89 ± 0.06	1.25 ± 0.06	m	1.90 ± 0.08	1.47 ± 0.14	m	2.03 ± 0.08	1.46 ± 0.10	m	2.05 ± 0.07	1.25 ± 0.15	m	1.95 ± 0.06	1.43 ± 0.08	m	1.68 ± 0.07	1.25 ± 0.09	m
8	1.76 ± 0.06	1.68 ± 0.23	sm	1.78 ± 0.06	1.11 ± 0.12	m	1.82 ± 0.06	1.17 ± 0.03	m	1.86 ± 0.05	1.36 ± 0.07	m	1.81 ± 0.08	1.42 ± 0.05	m	1.56 ± 0.05	1.19 ± 0.08	m
9	1.65 ± 0.04	1.11 ± 0.09	m	1.66 ± 0.04	1.18 ± 0.10	m	1.68 ± 0.04	1.07 ± 0.05	m	1.70 ± 0.04	1.16 ± 0.05	m	1.62 ± 0.06	1.23 ± 0.04	m	1.46 ± 0.04	1.14 ± 0.06	m
10	1.56 ± 0.04	1.50 ± 0.18	sm	1.52 ± 0.05	1.26 ± 0.15	m	1.54 ± 0.04	1.21 ± 0.05	m	1.56 ± 0.05	1.41 ± 0.11	m	1.47 ± 0.06	1.06 ± 0.04	m	1.36 ± 0.03	1.46 ± 0.14	m
11	1.52 ± 0.03	1.07 ± 0.05	m	1.44 ± 0.05	1.03 ± 0.02	M	1.48 ± 0.03	1.00 ± 0.00	M	1.46 ± 0.04	1.00 ± 0.00	M	1.38 ± 0.05	1.03 ± 0.05	m	1.32 ± 0.01	1.08 ± 0.07	m
∑	22.05 ± 0.71			22.55 ± 0.89			22.53 ± 0.65			23.24 ± 0.77			22.58 ± 0.89			20.04 ± 0.68		

Genomes sizes of species from Bisectae section, and *E. pulverulenta* from section Maidenaria were estimated by flow cytometry. These analyses also revealed very similar values among the species of *Eucalyptus* analyzed, with the average genome size values ranging from 1.25 and 1.26 pg/2C in *E. pulverulenta* and *E. cornuta*, respectively, and 1.27 pg/2C in *E. occidentalis* (Table 4). No statistically significant differences were observed among the species analyzed (*P* < 0.05).

**Table 4:** **Estimation of absolute DNA nuclear content in *Eucalyptus* species**.

**Species**	**Nuclear DNA content (pg/2C)**					
	**Mean**	**SD**	**CV (%)**	**Min**.	**Max**.	***n***
*Eucalyptus pulverulenta*	1.25	0.030	2.4	1.21	1.30	11
*Eucalyptus occidentalis*	1.27	0.039	3.1	1.21	1.32	11
*Eucalyptus cornuta*	1.26	0.017	1.3	1.23	1.28	10

*The values are given as mean and standard deviation (SD) of the nuclear DNA content (2C in picograms) of the individuals. Information of the CV value of the genome size estimations, as well as, of the minimum and maximum values is also provided. The number of analyzed individuals is also given. No statistically significant values at P < 0.05 were observed among species*.

**Figure 2:**
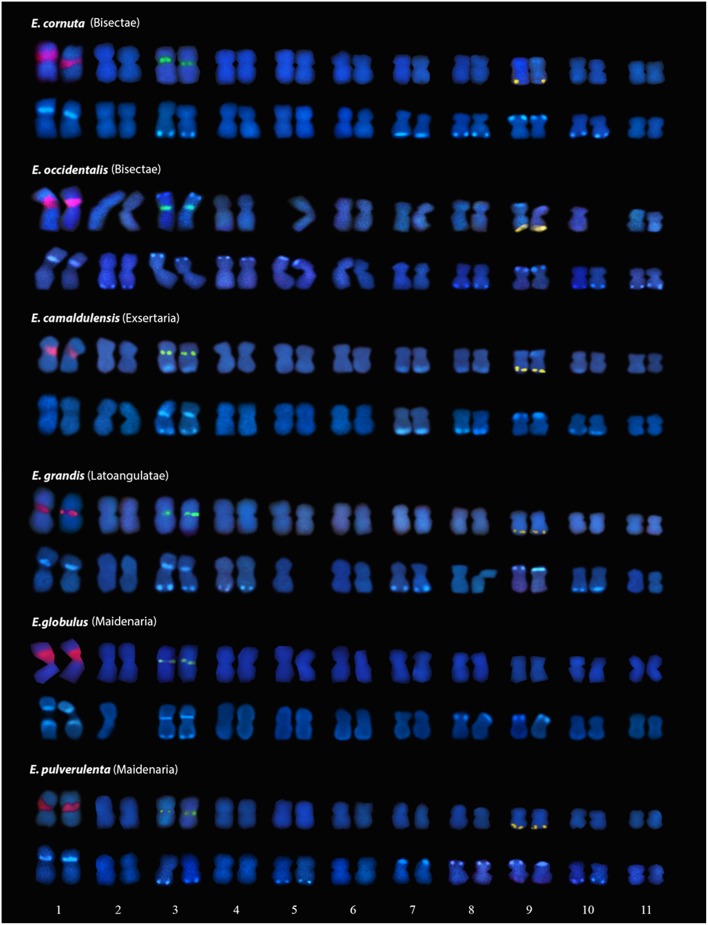
**Karyograms of *E. cornuta*; *E. occidentallis*; *E. camaldulensis*; *E. grandis*; *E. globulus*; *E. pulverulenta* with FISH markers (35S rDNA (first pair); 5S rDNA (third pair); BAC-CCR1 (ninth pair) above, and DAPI+ banding (lighter blue staining) below**. Missing homologous chromosomes were folded over itself in the analyzed metaphases and were unable to be used in these karyograms.

In order to map the rDNA loci, FISH with 35S rDNA, and 5S rDNA probes was performed in the six *Eucalyptus* species. All the species presented the 35S rDNA locus on the short arm of the first pair of chromosomes in a pericentromeric position leading to an evident secondary constriction. The 5S rDNA locus was located in the third pair of chromosomes, also positioned pericentromerically in the short arm (Figures 1, 2). Although the distribution pattern of the 35S rDNA was similar, the rDNA organization within the locus was different among species. The inner structure of each nucleolar organizer region (NOR) was clearly identified in the metaphase chromosomes by a large labeled condensed domain in the distal region present in all the species (Figure 1E inset), except from *E. grandis*. In this species apart from the large condensed site in the distal region, a small condensed site in the proximal region was detected (Figure 1D inset).

**Figure 3:**
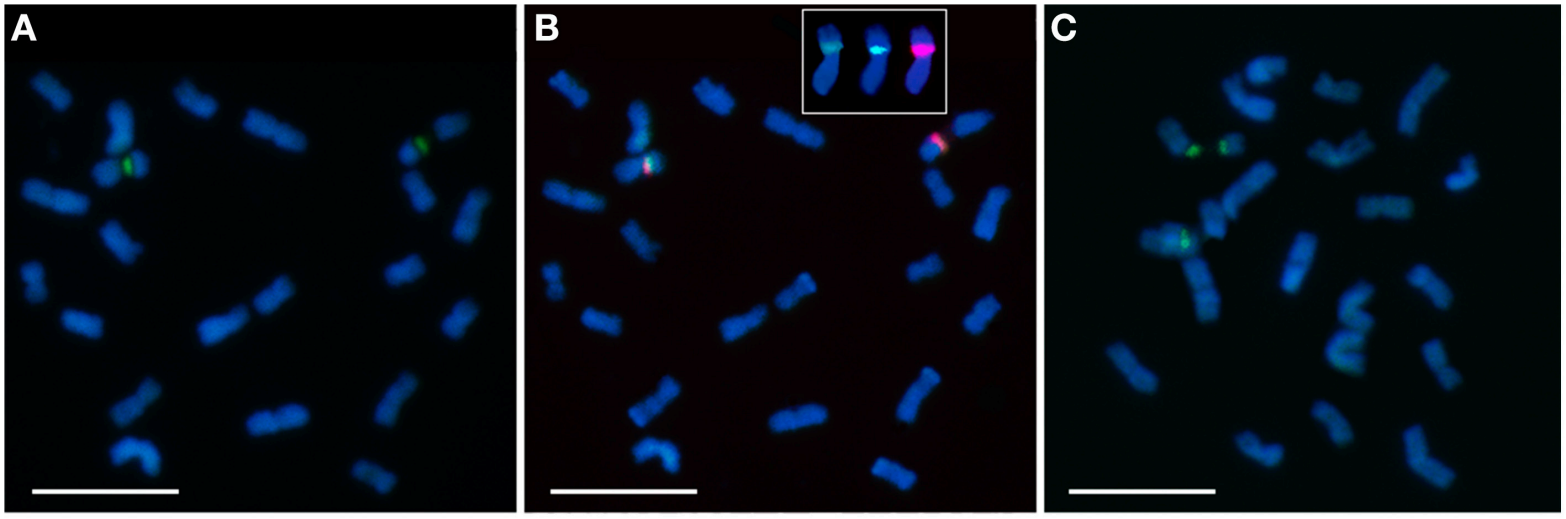
**Chromomycin A3 (CMA3) banding (green) in chromosomes of *E. cornuta* (A, B), representing the most common pattern, and of *E. grandis* (C)**. The CMA positive band is always co-localized with 35S rDNA (red/orange) **(B)** and with the DAPI+ in *E. grandis* (inset in **B**). Bar = 5 μm.

The presence of constitutive heterochromatin was evaluated through CMA3 and DAPI stainings. The GC-rich heterochromatin evaluated by CMA3 banding was restricted to the 35S rDNA loci in all species (Figures 3 and Supplementary Figure S1). The AT-rich blocks detected with the DAPI+ staining could be detected in the telomeric regions of various chromosomes in all species (Figure 2). Still, differences in number and distribution were observed among species, even of the same section, although in all the species studied there was one conserved DAPI+ block detected in the terminal region of the short arm of the nineth pair (Figure 2). *E. occidentalis* displays more DAPI+ bands, detected in nine chromosome pairs. The DAPI+ co-localization with CMA3 in the distal edge of the secondary constriction is also maintained in almost all species (Figure 3B inset), with the exception of *E. camaldulensis* (Figure 2). Furthermore, *E. globulus* presented DAPI+ blocks in both ends of the secondary constriction (Figure 2). *E. camaldulensis, E. grandis*, and *E. globulus* have a DAPI+ band co-localized with the 5S rDNA signal (Figure 2). In *E. occidentalis* the chromosome bearing 5S rDNA presented a DAPI+ band in the short arm, while in all other species, it was located at the end of the long arm (Figure 2).

### BAC landing of genes involved in lignin biosynthesis

**Figure 4:**
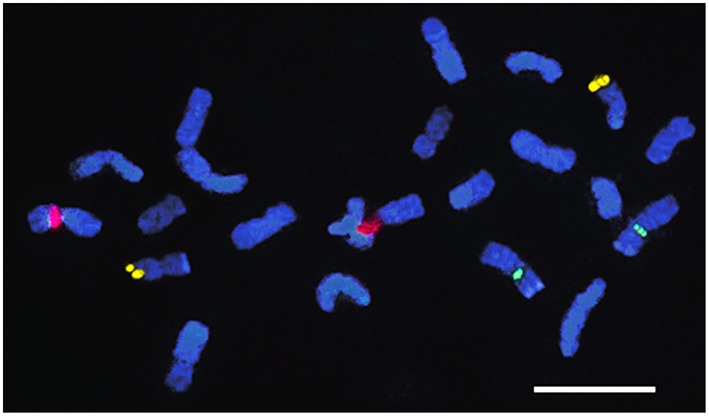
**Fluorescence *in situ* hybridization with BAC-CCR probe**. *E. occidentallis* metaphase chromosomes with 35S rDNA (red), 5S rDNA (green), and BAC-CCR (yellow) localizations. Bar = 5 μm.

**Figure 5:**
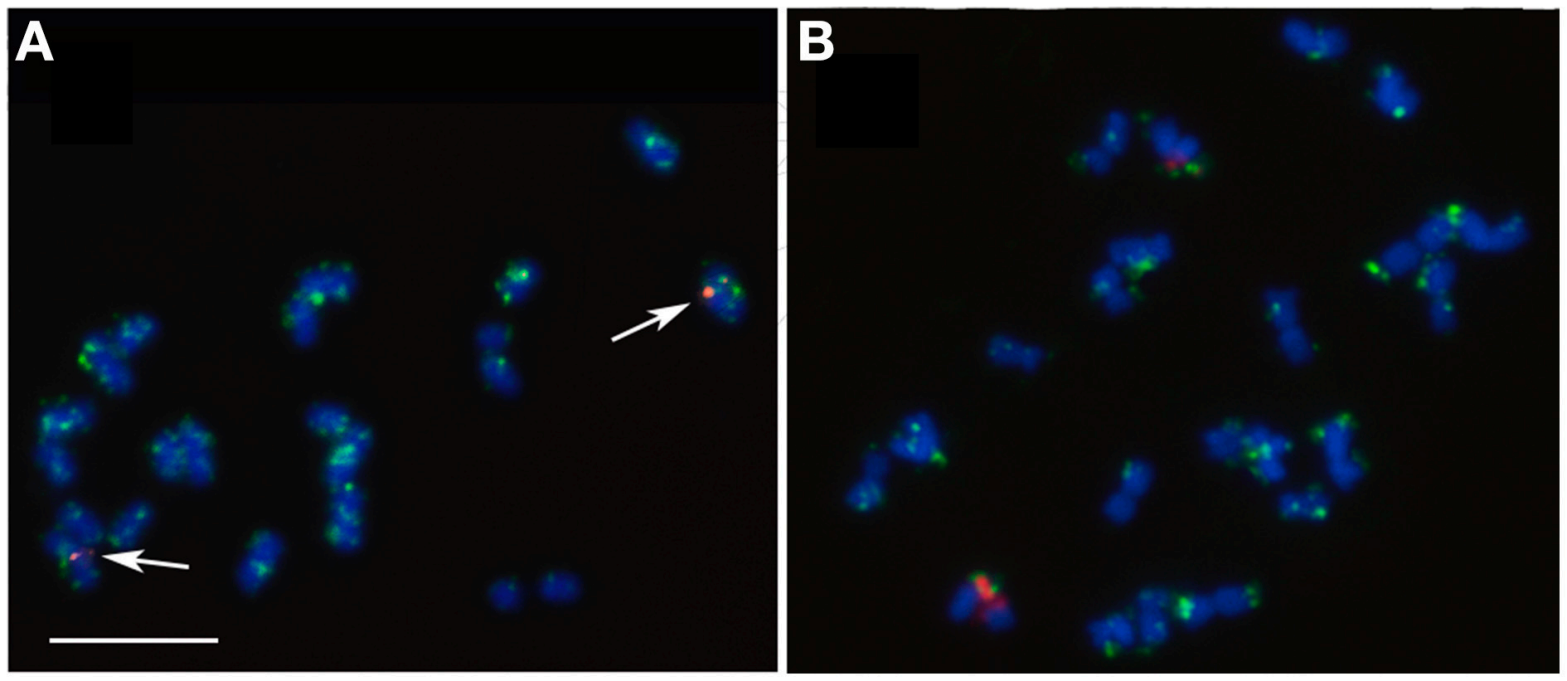
**Fluorescence *in situ* hybridization with BAC-RAC probe showing disperse pattern (green) along all chromosomes of *E. globulus* (A) and *E. grandis* (B)**. Co-localization (white arrows) with the 5S rDNA **(A)** and the 35S rDNA condensed and decondensed domains **(B)**. Bar = 5 μm.

**Figure 6:**
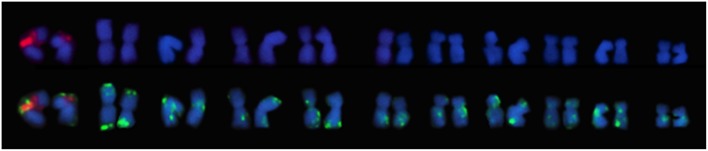
**Karyograms of *E. grandis* with 35S rDNA (red) and BAC-RAC1 probe (green) in chromosomes pairs (below) showing the co-localization of both probes in the first chromosome pair**.

FISH with two *E. globulus* BAC probes, EGC_Ba_P37H11 (BAC-CCR1) and EGC_Ba_P22O03 (BAC-RAC1), presented very distinct patterns. In all *Eucalyptus* species, BAC-CCR1 probe showed a single locus distal signal on the long arm of a small pair of chromosomes (Figure 4), which presents, in all species, terminal AT-rich blocks in the short arm (Figure 2). BAC-RAC1 probe hybridized in several locations along all the chromosomes of all species, in a disperse or in a cluster manner. Due to the dispersed nature of the signal it was not possible to precisely map the EglRAC1 gene locus (Figure 5). In all the studied species, the BAC-RAC1 signal was more represented in some domains of several chromosomes pairs such as telomeres, (peri)centromeres, and secondary constrictions co-localizing with the 35S rDNA and the 5S rDNA (Figure 5). A detailed analysis of *E. grandis* karyogram (Figure 6) allowed to precisely map the repetive sequences of BAC-RAC1 in this species: (a) in the first pair, the BAC-RAC1 was confined to the NOR region; (b) in the second pair was clustered at both telomeres; (c) the fourth and seventh pairs contained essentially pericentrometric signals; (d) the six and eleven pairs had dispersed signals only in the long arms, (e) finally, the nineth and tenth pairs had dispersed signal all along the chromosome arms.

## Discussion

### Comparative *Eucalyptus* karyotype analysis

Comparative karyotype analysis of six *Eucalyptus* species belonging to the subgenus Symphyomyrtus and to different sections, was achieved using chromosome morphology along with rDNA, BAC landing, and AT-rich as well as GC-rich DNA markers. These markers, together with genome sizes estimations, evidence a high conservation of the genomes within this subgenus.

The counting of chromosomes, especially, small ones, relying only on morphological comparison can lead to wrong conclusions like counting 2*n* = 24 (Matsumoto et al., 2000; Oudjehih and Abdellah, 2006), instead of the number fixed for the genus 2*n* = 22 (Grattapaglia et al., 2012). This error is often associated with counting pro-metaphases where the secondary constriction is much spread and the associated arm is very distant from the rest of the chromosome and can be counted as a very small chromosome. FISH with 35S rDNA marker can avoid this situation and even elucidate for pairs in the chromosome sets. FISH markers were essential for unequivocal establishment of chromosome pairs in this study.

Similar karyotype formulas of all species were evidenced, however, slight differences among species of the same section could be detected. Nevertheless, much more divergent karyotypes formulas, obtained exclusively from morphological data, were previously reported (Matsumoto et al., 2000; Mora et al., 2005; Cardemil and Perry, 2007). The small length of *Eucalyptus* chromosomes together with some differerences in condensation within chromosome pairs and the lack of unequivocal centromeric detection could be responsible for these differences in studies without additional chromosome markers. The *Eucalyptus* karyotypes studied are mainly composed of strictly metacentric (M) and median chromosomes (m), but submetacentric chromosomes (sm) appeared in *E. cornuta* and *E. occidentalis* from section Bisectae. These morphological differences could be due to chromosome rearrangements like deletions and amplifications or translocations of chromosome segments. However, the total chromosome lengths are very similar between species, and therefore, differences in chromosome morphologies rather than be due to large rearrangements seem to be dependent of local expansions of possible repetitive clusters. In fact, data from the *E. grandis* genome sequence has reinforced the idea that tandem duplications are an important mechanism in genome evolution of this species, since *E. grandis* has more tandem duplicates and more tandem expanded regions (clusters) than other plant genomes (Myburg et al., 2014).

Although some DAPI+ locations were conserved among species, DAPI banding patterns were unique to each *Eucalyptus* studied. This diversity may be caused by different redundancy levels of repeated DNA sequences. The differences between genome sizes of the *Eucalyptus* species are not significantly different. However, *E. occidentallis* shows a tendency to have a sigthly larger genome. This is in agreement with a larger amount of AT-rich constitutive heterochromatin, seen as DAPI+ blocks, in more chromosomes when compared with the other species studied. Bitonti et al. (1999) had already observed a positive correlation between genome size and the copy number of a given satellite DNA sequence in ten *Olea* species which was encountered in heterochromatin regions. Also, an example of intraspecific variation involving differences in heterochromatic sequences was described in the subspecies pair *Scilla bithynica* Boiss. ssp. *bithynica*, which presents many large C-bands, corresponding to constitutive heterochromatin, and a genome size of 29.2 pg and *S. bithynica* ssp. *radkae*, with few small C-bands and a genome size of 22.9 pg (Greilhuber, 1998).

Contrasting to the diversity of DAPI+ banding, we found the same rDNA pattern among the *Eucalyptus* species: one major pericentromeric 35S rDNA locus and one pericentromeric 5S rDNA locus in chromosome 1 and 3 respectively, evidencing genome conservation in species of the section Symphyomyrtus. However, we found a diferent organization of the 35S rDNA locus among these species: either only one condensed distal domain or two condensed domains in both ends of the NOR locus in *E. grandis*. These condensed domains, resulting from silent rDNA genes are usually defined as GC-rich constitutive heterochomatin (Guerra, 2000). The rDNA repeats are interspersed with the intergenic sequence (IGS) region, that is enriched in GC (Inácio et al., 2014), and therefore responsible for the CMA3+ staining at the NORs. Moreover, in almost all *Eucalyptus* species, a DAPI+ band co-localizes with the distal region of the 35S rDNA locus. The co-localization of these marks in the NOR is very rare, since the most common situation is the CMA3+ and DAPI- pattern (Guerra, 2000). This uncommon pattern also occures in *Hydrangea aspera* ssp. *sargetiana* (Mortreau et al., 2009) and it has been interpreted as ribosomal clusters interspersed with AT-rich heterochromatin (Colomba et al., 2006). The BAC-RAC1 probe which is enriched in several transposable elements (TEs), shows a strong signal hybridization in these domains. Moreover, high amount of pseudogenes from the 35S cistron with less GC content has been reported in several *Eucalyptus* genomes (Bayly et al., 2008). To explain the NOR organization we propose that more than one variant of rDNA repeats should be present in the 35S locus. The IGS lengths of some variants can be bigger due to the expansion of the AT-rich region as observed in Fagaceae (Inácio et al., 2014) which would be detected as a positive DAPI staining. Besides, the NOR condensed domains may be enriched in these pseudogenes which could be created by truncation of genes by TEs.

Notably, the 5S rDNA colocalizes with AT rich constitutive heterochromatin in species of different sections like *E. camaldulensis, E. grandis*, and *E. globulus*. Normally, this locus is DAPI neutral or could be in some species CMA3+ (Guerra, 2000). However, the same banding pattern was also reported in *Hydrangea aspera* from group Kawakami (Mortreau et al., 2009) and in *Maxillaria* species (Cabral et al., 2006). This could have several explanations: other unknown sequences with AT-rich regions may be intermingled, or around the 5S rDNA locus, or the proximity to transposable elements that tend to be silenced by heterochromatization. The association and proximity of transposable elements with genes is very clear in *Eucalyptus* genomes since the BAC-RAC1 probe is composed of several types of TEs in the vicinity of the gene RAC1 and remarkably, it is co-localized with the the 5S rDNA locus, besides the 35S rDNA. The presence of retroelements (RTEs) in NORs is an uncommon situation, but it has already been referred in the IGS of *Musa* sp. (Balint-Kurti et al., 2000), and in *Allium cernuum* (Chester et al., 2010). Also association with the 5S locus and the En/Spm transposon, has already been reported in Poaceae where clusters of this TE were found associated with heterochromatin (Altinkut et al., 2006). The expected silencing of these TEs in the NORs and 5S locus may therefore be a source for the DAPI+ staining in these regions. Besides the localization in NORs, 5S rDNA locus, and telomeres the BAC-RAC1 also showed a disperse signals along the chromosomes giving a pattern already detected in other plant species (Santos et al., 2015). Here we report the first work evaluating by FISH the distribution of repetitive DNA elements in *Eucalyptus*, and it is in agreement with the distributition of LTR RTE detected *in silico* (Marcon et al., 2015) where RTEs subfamilies were identified dispersed along chromosomes arms in both gene-rich and repetitive-rich regions. The localization of BAC-RAC1 on subtelomeric domains in several chromosomes agrees with the identification of *Copia* RTEs near the telomeric regions (Marcon et al., 2015).

### CCR1 gene location in *Eucalyptus* genomes

The assignment of the linkage groups (LGs) to individual chromosomes is of major importance for the integration of genetic and cytogenetic maps. Our integrated results unambigouysly localize the CCR1 gene into the terminal end of the long arm of the chromosome 9, although this gene has been previously assigned to LG10 (scaffold J) in *E. grandis* (Gion et al., 2015). It is well recognized that the correlation between chromosome size and genetic distances can sometimes be distorted. Whereas, LG are based on the assumption that crossover occurs at random along chromosomes, this is only partly true because genetic recombination is more likely to occur at some regions rather than at others (Han et al., 2011). Heterochromatic regions mostly enriched in RTEs are often associated with suppressed genetic recombination (Wang et al., 2006). *Eucalyptus* chromosomes 9 and 10 are highly enriched in disperse heterochromatin, detected by FISH with the BAC-RAC1, mainly composed of RTEs, and by DAPI+ staining. In this situation, the genetic recombination randomness along chromosomes may be affected, leading to the non correlation detected. In *Lotus japonicus*, where the distance calculated by recombination frequencies at the terminal regions is larger than the physical distance of the chromosomes this discrepancy is due to the presence of heterochromatin (Ohmido et al., 2010).

## Conclusions

In this work we mapped the rDNA loci, and the cinnamoyl CoA reductase gene, and determined the distribution of some retroelements in six species of *Eucalyptus*. We also calculated the genome size of three species. CCR1 gene was mapped into chromosome 9 in all species, clearly assign LG10 to physical chromosome 9.

A multi BAC-FISH approach with clones giving single-locus FISH signals is now mandatory to correctly identify *Eucalyptus* chromosomes, to support the established linkage groups and to better understand the evolution of *Eucalyptus* genus.

Our integrated results strengthened the conservation of the genomes in *Eucalyptus* species across four sections of subgenus Symphyomyrtus (Bisectae, Exsertaria, Latoangulatae, and Maidenaria). Thus, this work reinforces the idea that sequence information from the *E. grandis* genome can be transposed to other Symphyomyrtus species. This work is novel and contributes to the understanding of eucalypts genome organization which is essential to develop advanced breeding strategies for this genus.

## Author contributions

LMC and JP conceived the study and designed the experiments. TR, JL, and RB performed the experiments. TR, LM, and JP wrote the manuscript. HB and JP prepared the 3D BAC pools. CM collected the material and preformed the first analysis of the *E. globulus* BAC libraries. All authors read and approved the final manuscript.

### Conflict of interest statement

The authors declare that the research was conducted in the absence of any commercial or financial relationships that could be construed as a potential conflict of interest.

## Supplementary material

Supplementary Figure S1 **Metaphases of *E. occidentalis* (A), *E. camaldulensis* (B), *E. globulus* (C), and *E. pulverulenta* (D) with CMA3 green fluorescence in the NORs**. Bar = 5 μm.
