# Supplementary figures and images for: Advancing Eucalyptus Genomics: Cytogenomics Reveals Conservation of Eucalyptus Genomes
Source: Front Plant Sci. 2016 Apr 22;7:510. doi: 10.3389/fpls.2016.00510 (PMC4840385; doi:10.3389/fpls.2016.00510)

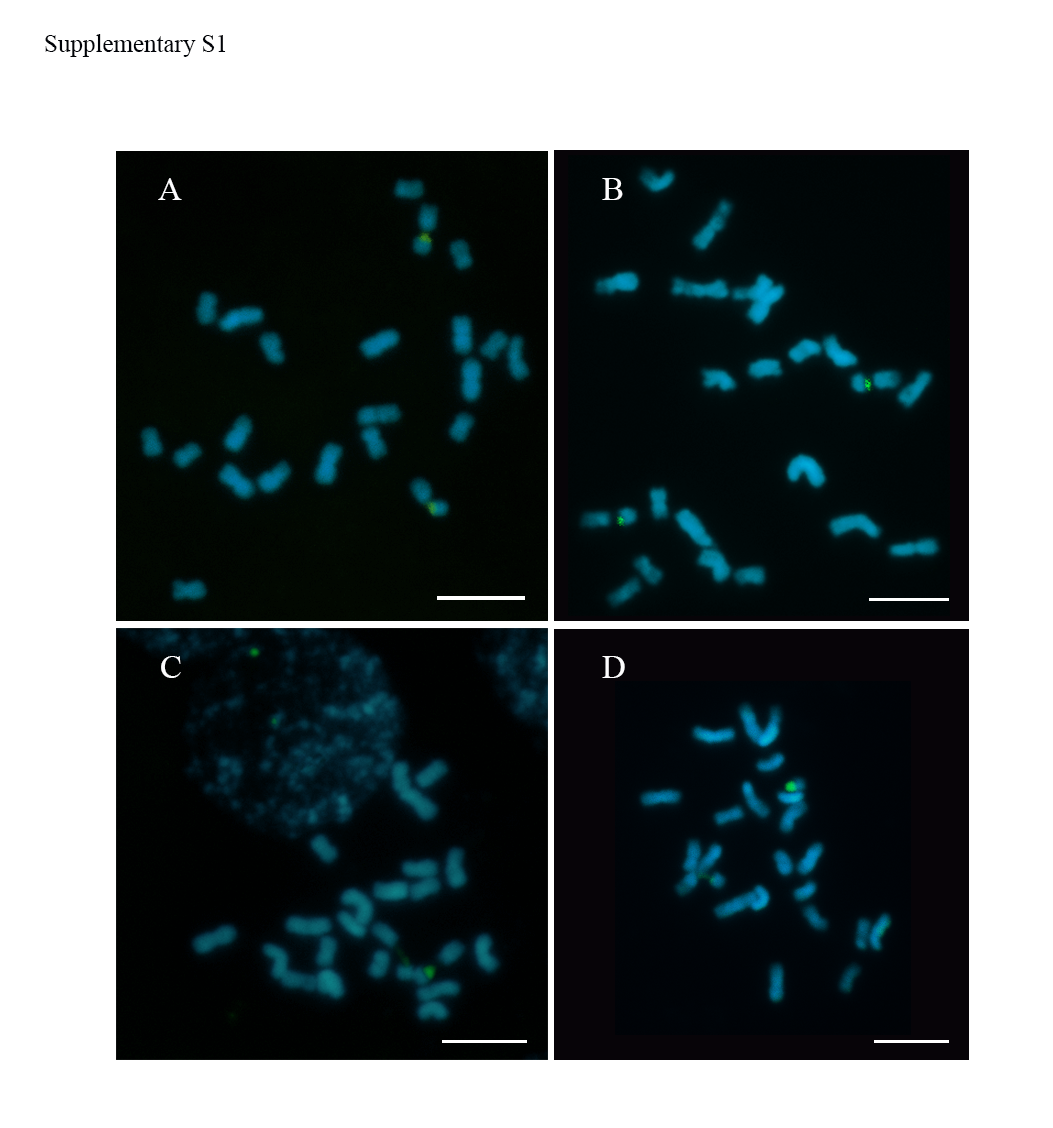

Supplement: Supplementary Figure S1 — Metaphases of E. occidentalis (A), E. camaldulensis (B), E. globulus (C), and E. pulverulenta (D) with CMA3 green fluorescence in the NORs. Bar = 5 μm. [file Image1.tif]
